# *Eudiplozoon nipponicum* (Monogenea, Diplozoidae) and its adaptation to haematophagy as revealed by transcriptome and secretome profiling

**DOI:** 10.1186/s12864-021-07589-z

**Published:** 2021-04-15

**Authors:** Jiří Vorel, Krystyna Cwiklinski, Pavel Roudnický, Jana Ilgová, Lucie Jedličková, John P. Dalton, Libor Mikeš, Milan Gelnar, Martin Kašný

**Affiliations:** 1 Department of Botany and Zoology, Faculty of Science, Masaryk University, Kotlářská 2, 611 37 Brno, Czech Republic; 2 Molecular Parasitology Laboratory, Centre for One Health, Ryan Institute, National University of Ireland Galway, Galway, Ireland; 3 Central European Institute of Technology, Masaryk University, Kamenice 5, 625 00 Brno, Czech Republic; 4 Department of Parasitology, Faculty of Science, Charles University, Viničná 7, 128 44 Prague, Czech Republic; 5 Department of Zoology and Fisheries, Centre of Infectious Animal Diseases, Faculty of Agrobiology, Food and Natural Resources, Czech University of Life Sciences Prague, Kamýcká 129, 165 00 Prague, Czech Republic

**Keywords:** *Eudiplozoon nipponicum*, Monogenea, NGS, Transcriptome, Assembly, Annotation, Secretome, Mass spectrometry

## Abstract

**Background:**

Ectoparasites from the family Diplozoidae (Platyhelminthes, Monogenea) belong to obligate haematophagous helminths of cyprinid fish. Current knowledge of these worms is for the most part limited to their morphological, phylogenetic, and population features. Information concerning the biochemical and molecular nature of physiological processes involved in host–parasite interaction, such as evasion of the immune system and its regulation, digestion of macromolecules, suppression of blood coagulation and inflammation, and effect on host tissue and physiology, is lacking. In this study, we report for the first time a comprehensive transcriptomic/secretome description of expressed genes and proteins secreted by the adult stage of *Eudiplozoon nipponicum* (Goto, 1891) Khotenovsky, 1985, an obligate sanguivorous monogenean which parasitises the gills of the common carp (*Cyprinus carpio*).

**Results:**

RNA-seq raw reads (324,941 Roche 454 and 149,697,864 Illumina) were generated, de novo assembled, and filtered into 37,062 protein-coding transcripts. For 19,644 (53.0%) of them, we determined their sequential homologues. In silico functional analysis of *E. nipponicum* RNA-seq data revealed numerous transcripts, pathways, and GO terms responsible for immunomodulation (inhibitors of proteolytic enzymes, CD59-like proteins, fatty acid binding proteins), feeding (proteolytic enzymes cathepsins B, D, L1, and L3), and development (fructose 1,6-bisphosphatase, ferritin, and annexin). LC-MS/MS spectrometry analysis identified 721 proteins secreted by *E. nipponicum* with predominantly immunomodulatory and anti-inflammatory functions (peptidyl-prolyl cis-trans isomerase, homolog to SmKK7, tetraspanin) and ability to digest host macromolecules (cathepsins B, D, L1).

**Conclusions:**

In this study, we integrated two high-throughput sequencing techniques, mass spectrometry analysis, and comprehensive bioinformatics approach in order to arrive at the first comprehensive description of monogenean transcriptome and secretome. Exploration of *E. nipponicum* transcriptome-related nucleotide sequences and translated and secreted proteins offer a better understanding of molecular biology and biochemistry of these, often neglected, organisms. It enabled us to report the essential physiological pathways and protein molecules involved in their interactions with the fish hosts.

**Supplementary Information:**

The online version contains supplementary material available at 10.1186/s12864-021-07589-z.

## Background

Monogeneans, typically ectoparasites of freshwater and marine fish, are the causative agents of major global fish diseases. Clinical symptoms of monogenean infections, such as tissue injury, anaemia, and respiratory and osmoregulatory dysfunctions, are often accompanied by secondary microbial infections [1, 2] that can lead to increased fish mortality. Monogenean infestations in fish aquacultures result in significant economic losses; in Norway, for example, annual economic losses due to the presence of the monogenean *Gyrodactylus salaris* in commercial breeding stocks of Atlantic salmon (*Salmo salar*) are estimated at over 500 million USD [3].

Despite the economic importance of this group of ectoparasites, there is a shortage of data on their functional molecular biology and interactions with their fish hosts. To reveal phylogenetic relationships within the species-rich monogenean group, several studies focused on sequencing the mitochondrial genome. This research investigated, for instance, (a) polytypic parasites *G. salaris* [4], which infects salmon, and *Gyrodactylus thymalli* [5], which typically infect the grayling; (b) *Neobenedenia melleni*, a generalist parasite of marine fish [6]; (c) *Benedenia hoshinai* [7], a parasite of the striped knifejaw *Oplegnathus fasciatus*, and (d) *Pseudochauhanea macrorchis* [8], which typically infects the pickhandle barracuda *Sphyraena jelloi*.

To date, however, only three monogenean genomes are publicly available, namely the genome of *G. salaris* [9], *Gyrodactylus bullatarudis*, an ectoparasite of guppy fish *Poecilia reticulata* [10], and *Protopolystoma xenopodis*, which infects the African clawed frog *Xenopus laevis* [11]. *G. salaris, G. bullatarudis*, and *P. xenopodis* represent phylogenetically different monogenean subclasses (Monopisthocotylea and Polyopisthocotylea) with significant differences in their genome size (67.38 Mb, 84.40 Mb, and 617.34 Mb respectively) and the number of coding genes (15,436, 10,749, and 37,906). Other publicly available datasets include an EST dataset containing 6726 sequences for *N. melleni* (unpublished, NCBI BioSample SAMN00169373) and a recent proteomic study targeting tissue-specific proteins of the adult stage of *E. nipponicum* [12].

*E. nipponicum* is an oviparous, blood-feeding ectoparasite which infests the gills of the common carp. During its unique lifecycle, two larvae (diporpae, post-oncomiracidial stage) permanently fuse to form the juvenile stage, which then develops into an adult individual [13, 14]. *E. nipponicum* was introduced to Europe from Southeast Asia prior to 1983 [15] and has since become a common parasite of carp with negative impact on their populations [16]. It presents a particular problem for intensive pond carp farming in Europe, which produces over 187,000 tons of carp a year (based on data from 2018) [17].

Despite the economic importance of *E. nipponicum*, only a handful of studies so far investigated the genes of this parasite, so that to date, only 38 nucleotide and 10 amino acid sequences are deposited in NCBI databases. Key studies focused on (a) genetics and molecular biology [16, 18–26], in particular molecular identification and characterisation of key peptidases and their inhibitors, namely cathepsins L, B, and D [21, 22], cystatin [20], serpin [23], and a Kunitz-type inhibitor [24]; (b) cytogenetics [27] and phylogenetics, with the aim to further our understanding of monogenean evolution; (c) morphological adaptations to ectoparasitism [13, 14, 28]; (d) involvement of surface carbohydrates [29] during the fusion process between diporpae and in interaction with the fish host, and finally, (e) the effect of somatic fusion between the two diporpae on the neural system [30–32]. In the present study, we report and make publicly available the first global investigation into the biology of *E. nipponicum* using an integrated transcriptomic and proteomic approach and escribe certain important new aspects of the ectoparasite–host relationship.

## Results

### Transcriptional profile of adult *E. nipponicum*

A total of 324,941 Roche 454 raw reads (length 424 ± 219 bp) and 149,697,864 Illumina raw reads (length 100 bp) were processed, assembled, and merged into 94,814 contigs. The contigs were clustered and filtered, resulting in 37,062 protein-coding transcripts with mean length of 736 bp, which were used for subsequent analysis (Table 1; Additional file 1: Table S1). The transcripts were annotated using seven databases, resulting in 53% (*n* = 19,644) of transcripts annotated by at least one database (Table 2; Additional file 2: Table S2).

**Table 1:** Statistics of raw reads and assembled transcripts from Roche 454 and Illumina reads

Basic statistics of raw reads	
Total number of obtained raw reads	150,022,805
Illumina MiSeq	149,697,864
Roche 454	324,941
Average length of obtained raw reads	
Illumina MiSeq	100 bp
Roche 454	424 ± 219 bp
Total number of processed reads before assembly	123,774,558
Illumina MiSeq	123,555,644
Roche 454	218,914
Statistics of transcriptome assembly	
Total number of final transcripts	37,062
Mean length of nucleotide sequences	736 bp
Number of transcripts encoding full-length protein ≥30 amino acids	14,203 (38.32%)
Number of transcripts with start codon (ATG) only	3689 (9.95%)
Number of transcripts terminated by a stop codon only	13,092 (35.32%)
Complete/partial matches to 248 CEGMA core proteins	69.35, 90.73%
978 searched BUSCO groups (%)	Comp: 776 (79.34) [Single 436 (44.58), Dupl: 340 (34.76)], Fragm: 63 (6.44), Missing: 139 (14.21)
GC content in transcripts	42.30%
N50	1548 bp
N90	360 bp

**Table 2:** Summary of annotation results of the *E. nipponicum* transcriptome

Transcriptome annotation	
Total number of annotated transcripts	19,644 (53.0%)
Number of transcripts homologue to nucleotide DDBJ database	1342 (3.62%)
Number of proteins homologous to entries in protein used databases	
NCBI non-redundant (nr) protein database	1533 (4.14%)
RCSB PDB	28 (0.08%)
UniProtKB/Swiss-Prot	131 (0.35%)
UniProtKB/UniRef100	1886 (5.09%)
UniProtKB/TrEMBL (phylum Platyhelminthes only)	14,020 (37.83%)
MEROPS peptidases	555 (1.50%)
MEROPS peptidase inhibitors	149 (0.40%)
Number of transcripts with assigned KO number	7435 (20.06%)
Number of unique KO numbers	3499
Number of transcripts classified by InterProScan	16,772 (45.25%)
Number of transcripts with assigned GO term/s	11,149 (30.08%)
Number of unique GO terms	1627
Biological process	640
Cellular component	267
Molecular function	720
Analysis of excretory-secretory proteins	
Number of ESP identified by mass spectrometry	721 (1.95%)

Analysis of transcript abundance revealed that adult *E. nipponicum* parasites are transcriptionally active, with the top 100 transcripts representing approximately 39% of total transcription, represented by TPM (Additional file 2: Table S2). Within these abundantly expressed transcripts, uncharacterised proteins prevail. Among the annotated transcripts, ribosome-associated and ubiquitin-related transcripts are highly transcribed. Consistently with the haematophagous strategy used by *E. nipponicum*, key genes associated with blood feeding and digestion also belong to the most transcribed: they include a number of ferritins (iron storage proteins), Kunitz-type inhibitor KT1 (anticoagulation properties), and a CD59-like transcript (inhibition of the complement cascade) (Additional file 3: Table S3).

**Fig. 1:**
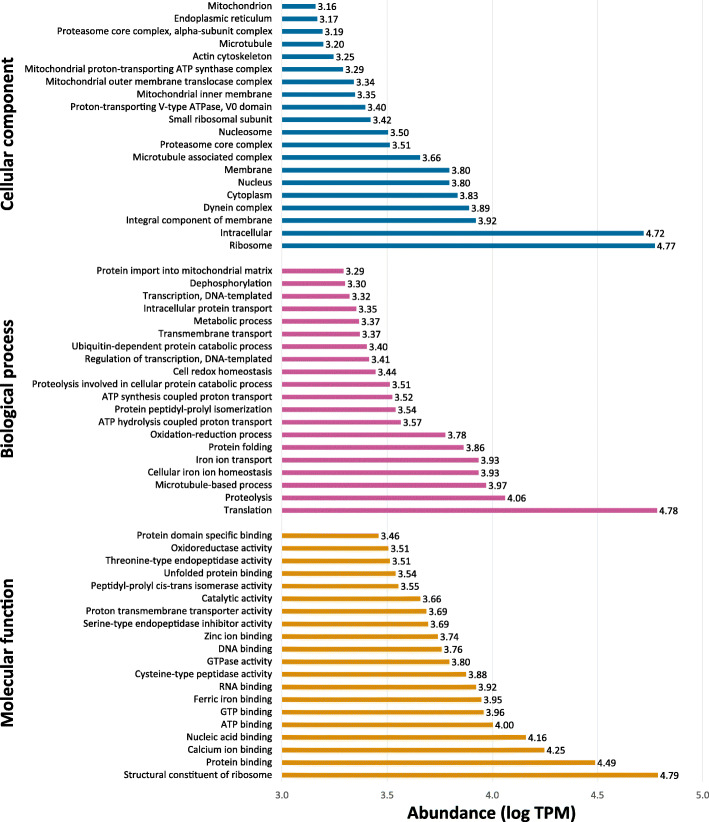
GO term distribution among *E. nipponicum* transcripts. The most expressed GO terms, top 20 for each main category (in ascending order, axis y), and their distribution in the three main GO categories: Cellular component (blue), Biological process (purple), and Molecular function (orange). Expression level (axis x) is based on the sum of TPM values for all transcripts included for each GO term. A logarithmic scale was used to display the relative expression of each GO term

Analysis by gene ontology (GO) of the total transcriptome revealed that abundant transcription is associated with GO terms related to binding, protein synthesis, and catalytic activity. In particular, key GO terms associated with the ribosome (GO:0003735, TPM: 61,254.60, ratio of TPM values and number of associated transcripts (referred to herein as TPM/transcript ratio): 189.64; GO:0005840, TPM: 59,162.10, TPM/transcript ratio: 202.61) and with proteolysis (GO:0006508, TPM: 11,499.80, TPM/transcript ratio: 35.94) were among the most expressed (Fig. 1; Additional file 4: Table S4). Abundant transcription of GO terms related to iron and haem processing was also observed; this included ferric-iron binding (GO:0008199, TPM: 8865.16, TPM/transcript ratio: 227.31), cellular iron ion homeostasis (GO:0006879, TPM: 8594.39, TPM/transcript ratio: 245.55), iron ion transport (GO:0006826, TPM: 8593.63, TPM/transcript ratio: 306.92), iron ion binding (GO:0005506, TPM: 560.70, TPM/transcript ratio: 23.36), and haem binding (GO:0020037, TPM: 2419.98, TPM/transcript ratio: 96.80).

A recent in-depth analysis of proteins secreted by the *E. nipponicum* had shown that extracellular vesicles (EVs) play a key role at the host–parasite interface. In particular, they facilitate infection and parasite survival [33]. The role of extracellular vesicles in relation to monogenean parasites is yet to be explained but our analysis of adult *E. nipponicum* transcriptome identified a number of transcripts associated with extracellular vesicular transport, which indicates that EVs might play a role in interactions between the host and *E. nipponicum*. In particular, transcripts associated with the KEGG exosome term (ko04147, TPM: 38,549.90, TPM/transcript ratio: 57.45) are abundantly expressed and, consistently with the KEGG analysis, GO terms related to endocytosis (GO:0006897, TPM: 42.02), vesicle docking (GO:0048278, TPM: 17.35), vesicle-mediated transport (GO:0016192, TPM: 1263.80, TPM/transcript ratio: 10.44), and vesicle docking involved in exocytosis (GO:0006904, TPM: 100.90, TPM/transcript ratio: 6.73) were also observed.

### Analysis of *E. nipponicum* metabolism

**Fig. 2:**
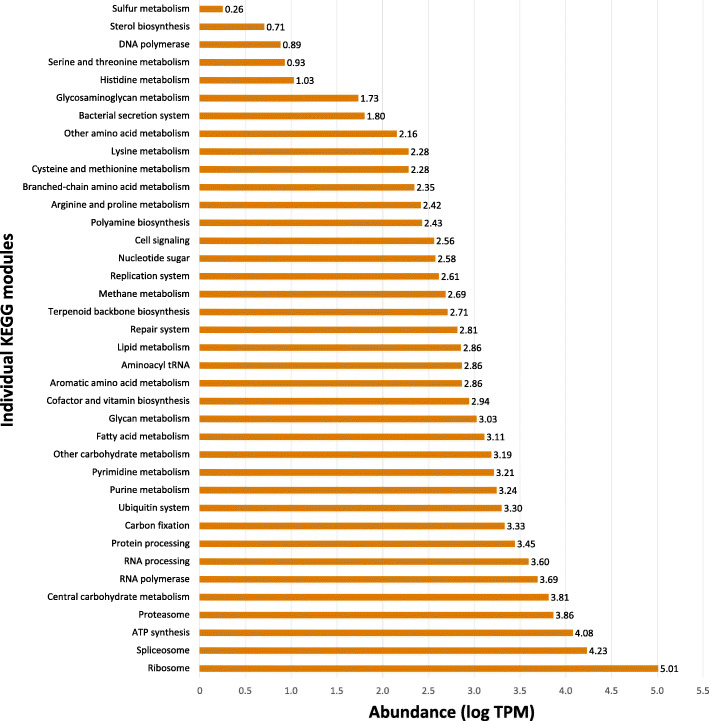
Abundance of *E. nipponicum* KEGG modules. A list of all observed KEGG modules [34] (axis y) in *E. nipponicum* transcriptome sorted (in ascending order) according to their expression level based on the sum of TPM values for all transcripts included in each module (axis x). A logarithmic scale was used to display the relative expression of each KEGG module

**Fig. 3:**
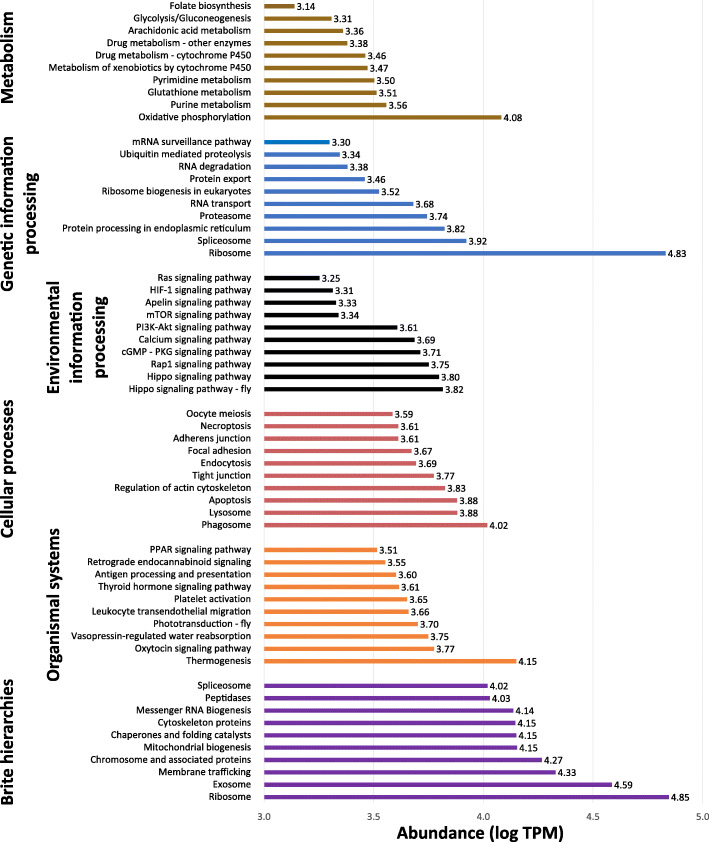
Assignment of the *E. nipponicum* transcripts to individual KEGG Orthology categories. KEGG pathways [34] (axis y, top 10 for each category) sorted (in ascending order) by their abundance (expressed by the sum of TPM values for all included transcripts, axis x) in the main categories: brown (A09100 Metabolism), blue (A09120 Genetic information processing), black (A09130 Environmental information processing), red (A09140 Cellular processes), orange (A09150 Organismal systems), and purple (A09180 Brite hierarchies). Categories A09160 (Human diseases) and A09190 (Not included in pathway or brite) were excluded. A logarithmic scale was used to display the relative expression of each pathway

There is as yet a paucity of data regarding the type of energy metabolism used by *E. nipponicum*, but because the parasite lives in an oxygen-rich environment on carp gills, one can safely assume that its metabolism is predominantly aerobic. This hypothesis is supported by KEGG metabolic pathway analysis, which revealed a transcription of genes associated with the glycolysis and gluconeogenesis pathway (ko00010, TPM: 2028.11, TPM/transcript ratio: 35.58), citrate cycle (ko00020, TPM: 866.91, TPM/transcript ratio: 19.70) and oxidative phosphorylation (ko00190, TPM: 12,081.56, TPM/transcript ratio: 67.87), all of which are indicative of aerobic metabolism (Figs. 2 and 3; Additional File 5: Table S5; Additional File 6: Table S6).

An analysis of enzymes involved in glycogen synthesis and catabolism revealed low levels of transcription of phosphofructokinase (related to glycogen breakdown; 4.30 TPM), which is in keeping with the fact that in oxygen-rich environment, *E. nipponicum* energy metabolism relies on its glycogen stores. In comparison, a higher transcription was observed for an enzyme involved in glycogen synthesis, namely fructose 1,6-bisphosphatase (86.34 TPM), which may be required for vitelline cell development during the parasite’s egg formation process [35].

Other key pathways with high levels of transcription included pathways associated with signal transduction, which regulate a range of cell function processes and play a critical role in cellular development (Fig. 3). Among the KEGG pathways transcribed, we also observed the digestion process. Ingested blood is partially hydrolysed in the gut under slightly acidic conditions (gastric acid secretion ko04971, TPM: 1443.04, TPM/transcript ratio: 34.36) and released molecules (proteins, fats, carbohydrates, and vitamins) are processed intracellularly within digestive cells (protein digestion and absorption ko04974, TPM: 375.11, TPM/transcript ratio: 15.63; fat digestion and absorption ko04975, TPM: 278.19, TPM/transcript ratio: 14.64; carbohydrate digestion and absorption ko04973, TPM: 196.92, TPM/transcript ratio: 7.88; vitamin digestion and absorption ko04977, TPM: 27.62) before being absorbed (phagosome ko04145, TPM: 10,427.96, TPM/transcript ratio: 68.61; endocytosis ko04144, TPM: 4931.40, TPM/transcript ratio: 26.95).

### Abundant transcription of peptidases and peptidase inhibitors

Consistently with recent somatic proteomic analysis of adult *E. nipponicum* by Roudnický and colleagues [12], peptidases and their inhibitors are highly transcribed in the adult transcriptome data. Transcripts associated with the GO term peptidase (GO:0008233, TPM: 1561.66, TPM/transcript ratio: 82.19) and endopeptidase (GO:0004175, TPM: 2693.81, TPM/transcript ratio: 99.77) are significantly transcribed and dominate the five main classes of peptidase activity: cysteine-type peptidase activity (GO:0008234, TPM: 7500.09, TPM/transcript ratio: 83.33), threonine-type endopeptidase activity (GO:0004298, TPM: 3257.16, TPM/transcript ratio: 70.81), serine-type endopeptidase activity (GO:0004252, TPM: 1080.0, TPM/transcript ratio: 15.65), aspartic-type endopeptidase activity (GO:0004190, TPM: 1050.0, TPM/transcript ratio: 19.44), and metalloendopeptidase activity (GO:0004222, TPM: 419.22, TPM/transcript ratio: 8.56). Key GO terms associated with endopeptidase inhibitory activity also displayed abundant transcription within the adult transcriptome (GO:0004867 serine-type endopeptidase inhibitors, TPM: 4950.51, TPM/transcript ratio: 72.80 and GO:0004866 endopeptidase inhibitor activity, TPM: 664.42, TPM/transcript ratio: 44.29).

Further in-depth analysis of these transcripts that used the MEROPS peptidase database identified 555 proteases and 149 inhibitors, which were classified into 62 peptidase and 15 inhibitor families (Table 3).

**Table 3:** *E. nipponicum* peptidases and inhibitors divided in individual catalytic types according to the MEROPS database

Catalytic type	Transcripts/TPM	Families
Aspartic peptidases	35/226.10	4
Cysteine peptidases	187/1586.49	22
Metallo peptidases	166/2169.10	21
Serine peptidases	121/1849.18	12
Threonine peptidases	46/2434.21	3
Peptidase inhibitors	149/5473.71	15

Peptidase classification was consistent with the GO analysis, reflecting a predominance of threonine, metallo, serine, and cysteine peptidases (Fig. 1). Of the three peptidase families with threonine peptidase activity, the most abundantly transcribed genes belong to proteasome-related threonine T1 family (PB clan), which reflects intensive protein turnover during this parasite stage. Supporting the protein degradation role played by the proteasome, our analysis shows that genes associated with the metallo peptidase family M67, which plays a critical role in deubiquitination of proteins, are also highly transcribed. Abundant transcription of genes associated with families Serine S1 (chymotrypsin family; PA clan) and Cysteine C2 (calpain family; CA clan) similarly support critical processes such as development and digestion [36] (family Serine S1), as well as signal transduction, cellular differentiation, cytoskeletal remodelling, and vesicular trafficking (family Cysteine C2) [37].

Analysis of the 15 peptidase inhibitory families showed a predominance of inhibitors of serine and cysteine peptidases, specifically inhibitors belonging to family I29, which consists of inhibitors of C1 papain-like cysteine peptidases (Table 4). This is consistent with our previous biochemical characterisation of three key peptidase inhibitors highly expressed in the adult parasite secretome, namely a type I cysteine peptidase inhibitor (EnStef) [20] and two inhibitors of serine peptidases, namely serpin EnSerp1 [23] and Kunitz-type inhibitor EnKT1 [24].

**Table 4:** Transcripts of *E. nipponicum* related to families of peptidase inhibitors according to the MEROPS database

Family	Transcripts/TPM	Target peptidases
I1	6/5.46	**Serine endopeptidases**, referred to as Kazal family inhibitors, inhibit peptidases from families S1 (chymotrypsin) and S8 (subtilisin)
I2	21/79.43	**Serine peptidases** inhibit chymotrypsin peptidases (S1 family) and include trypsin, chymotrypsin, tissue kallikrein, and plasmin
I4	9/449.59	**Serine** and **cysteine endopeptidases** inhibit families S1 (chymotrypsin), S8 (subtilisin), C1 (papain), and C14 (caspase)
I8	3/134.44	**Serine** and **metallo endopeptidases** target peptidases belonging to families S1 and M4 (thermolysin)
I15	3/68.58	**Serine endopeptidases** from the chymotrypsin (S1) family
I21	3/210.24	**Serine endopeptidases** from the subtilisin (S8) family
I25	1/16.40	Primarily papain-like (C1 family) **cysteine peptidases**
I29	25/2698.10	Papain-like (C1 family) **cysteine peptidases**
I32	9/7.85	Caspases, **cysteine endopeptidases** from family C14
I39	29/235.98	**Endopeptidases regardless of their catalytic type**
I43	2/1.44	**Metallo peptidases** from the astacin/adamalysin (M12) family
I51	3/87.27	**Serine carboxypeptidase Y** (S10.001)
I63	9/302.97	**Metallo peptidase pappalysin-1** (M43.004)
I87	20/1169.99	**FtsH metallo peptidase** (M41.001)
I93	6/5.97	**Metallo peptidases**, especially M12 family (astacin family)

### Key *E. nipponicum* molecules important for blood feeding in adult stage parasites

Members of clade Neodermata synthesise haem via haem biosynthesis pathway consisting of eight enzymatic steps [38–40]. *E. nipponicum*, like other haematophagous parasites which after adopting a blood feeding strategy lost the ability to synthesise haem de novo, relies during egg production solely on host blood as a rich source of carbohydrates for energy metabolism, amino acids, and fatty acids. Still, physiological haem plays a critical role in a wide range of this parasite’s biological processes [41]. Interestingly – and in contrast to many blood-feeding parasites that lost most enzymes belonging to the haem biosynthesis pathway – homologues of seven such enzymes have been identified in *E. nipponicum* transcriptome (Table 5).

**Table 5:** Proteins of *E. nipponicum* participating in the haem synthesis pathway

Enzyme	Transcripts/TPM	Function
5-Aminolevulinic acid synthase (ALAS)	No hit	Formation of 5-aminolevulinic acid (ALA) by condensation of succinyl-CoA and glycine in the mitochondria
5-Aminolevulinic acid dehydratase (ALAD)	2/31.61	Formation of porphobilinogen (PBG) from ALA after exporting to the cytosol
Porphobilinogen deaminase (PBGD)	1/8.90	Condensation of four PBGs to the unstable hydroxymethylbilane (HMB)
Uroporphyrinogen III synthase (UROS)	1/13.46	Cyclisation of HMB to uroporphyrinogen III (URO III)
Uroporphyrinogen III decarboxylase (UROD)	3/55.85	Decarboxylation of four acetic side chains to methyl groups to form coproporphyrinogen III (COPRO III)
Coproporphyrinogen III oxidase (CPO)	1/12.32	Formation of protoporphyrinogen IX (PP’gen IX) back in the mitochondria
Protoporphyrinogen oxidase (PPO)	2/14.95	Oxidation of PP’gen IX yielding protoporphyrin IX (PP IX)
Ferrochelatase (FC)	2/5.11	Catalyse the insertion of a ferrous ion into PP IX, creation of the final product (the haem)

In fact, only the transcript for 5-Aminolevulinic acid synthase (ALAS), enzyme responsible for initiation of the pathway, was absent. Further in-depth genomic analysis is required to confirm the absence of this crucial gene and to determine whether this is a feature which *E. nipponicum* shared with other haematophagous monogeneans. Similarly, functional analysis of the seven identified homologous enzymes would determine whether these enzymes evolved other functions important for the parasite.

Based on the results of structural and histochemical analyses [42, 43], one can conclude that the blood digestion process in *E. nipponicum* resembles intracellular digestion that takes place inside digestive cells and is usually found in ectoparasitic haematophagous mites, such as ticks. Nevertheless, a recent study [21] seems to indicate that an extracellular phase of digestion, in the lumen of the gut, is also present and blood digestion in *E. nipponicum* thus more closely resembles digestive processes in liver flukes [44] rather than in ticks. Erythrocytes are probably lysed within the monogenean gut lumen, releasing haemoglobin tetramers which are then hydrolysed in specialised haematin cells of the phagolysosome [45]. This process releases iron-rich haem which plays an important role in a number of biological processes [41] and is a crucial component required for egg production [46]. To protect the parasite from haem toxicity and related effects of oxidative stress, iron ions are stored in intracellular iron storage proteins, ferritins [47]. Analysis of *E. nipponicum* transcriptome shows that ferritins are amongst the most transcribed genes, represented by 36 transcripts (14,024.50 TPM). This finding is consistent with studies by Galay and colleagues which show that multiple ferritins are critical for successful blood feeding and reproduction in hard ticks [48].

Residual free haem is removed by conversion to haematin crystals which are expelled back into the gut lumen and regurgitated by the worm into the outer environment [45]. Similarly to the nematodes and the tick *Ixodes ricinus* [49]*, E. nipponicum* does not encode a gene for haem oxygenase. The process of haem detoxification by catabolism is therefore mediated by high affinity haem-binding proteins, glutathione S-transferases (GSTs) [49, 50], which are abundantly transcribed within the *E. nipponicum* transcriptome. We identified 29 transcripts, including 24 mu class and two mitochondrial kappa class GSTs (3554.0 TPM).

Cathepsin cysteine peptidases are essential for degradation of host haemoglobin and are abundantly transcribed in the *E. nipponicum* transcriptome. Consistent with a study by Jedličková and colleagues [21], adult *E. nipponicum* transcribe mainly cathepsin L peptidases, specifically cathepsin L1 and L3 (*n* = 36; 3789.31 TPM) at a ratio of 29 cathepsin L1 (major transcript of cathepsin L1 = 969.02 TPM) to five cathepsin L3s (major transcript of cathepsin L3 = 515.64 TPM) and two unspecified cathepsins L-like, while the expression of cathepsin B peptidases is lower (*n* = 4; 468.82 TPM).

We also found a number of transcripts encoding calcium-dependent, non-lysosomal calpain-like proteases (*n* = 10; 354.97 TPM), which cleave the blood-clotting fibronectin and thereby facilitate parasite feeding [37, 51]. Cathepsin D aspartic endopeptidase (*n* = 5; 314.49 TPM) and aminopeptidases that use a metal ligand within their active site (*n* = 12; 103.12 TPM), such as aminopeptidase P3 (also known as Xaa-Pro aminopeptidase) and aminopeptidase A (or glutamyl aminopeptidase), also probably play a role in blood digestion, although not to the same extent as similar molecules in other haematophagous ectoparasites, such as ticks.

Our biochemical characterisation of key secreted peptidase inhibitors had shown that they play a critical role during blood feeding [20, 23, 24]. Kunitz-type inhibitor KT1 is among the most abundantly transcribed genes of the *E. nipponicum* transcriptome (*n* = 68; 16,683.0 TPM). This interesting serine protease inhibitor has anticoagulation properties and can impair the host complement system [24]. The adult parasite also transcribes other serine protease inhibitors, especially serpins (*n* = 20; 414.49 TPM), though at lower levels of transcription. These serpins have been shown to play a role in the suppression of blood coagulation (by targeting mainly factor Xa), complement activation, and fibrinolysis [23]. Similarly, we identified a number of transcripts encoding a type I cysteine peptidase inhibitor (cystatin/stefin; n = 6; 574.20 TPM), which has been shown to be involved in the regulation of haemoglobin degradation [20].

### Proteins probably acting at the host–parasite interface

To date, only a handful of proteomic studies have been carried out for *E. nipponicum*, namely a gel-based analysis of secreted proteins that focused on cathepsin peptidases [21, 22] and a study of microdissected tissue-specific somatic proteins [12]. In our study, we conducted a gel-free proteomic analysis of excreted–secreted proteins (ESP), otherwise known as secretome of adult *E. nipponicum*, which identified 721 proteins with at least two unique peptides (Additional file 7: Table S7). Consistent with the transcriptome analysis, most of these proteins have not been characterised previously. We identified several key proteins involved in blood feeding and digestion, namely a number of ferritins (*n* = 7; 1.25 NSAF) and glutathione S-transferases (*n* = 7; 1.95 NSAF), which are abundantly transcribed in the adult transcriptome and consequently also abundantly expressed.

We have previously reported that cathepsin L peptidases dominate the proteolytic activity of adult *E. nipponicum* secretome, where they in conjunction with cathepsin B peptidases play a critical role in haemoglobin degradation. Both these peptidases were identified in our current secretome analysis (cathepsin L1: 1.26 NSAF; cathepsin B: 0.14 NSAF). Additionally, we observed other key peptidases involved in blood digestion, including (a) calpain, which cleaves the blood-clotting protein fibronectin and therefore has an anticoagulation effect (0.19 NSFA) [51]; (b) aspartic endopeptidase cathepsin D (0.04 NSFA), which probably cleaves haemoglobin [52]; (c) saposin, involved in red cell lysis (0.01 NSFA) [53]; and (d) cathepsin C, also known as dipeptidyl peptidase I (0.13 NSFA), which in schistosomes may in conjunction with leucine aminopeptidases play a role in the terminal hydrolysis of haemoglobin-derived peptides [54].

Despite the high levels of gene transcription, the Kunitz-type inhibitor KT1 previously characterised by us [24] was not highly abundant in the secretome (0.02 NSAF). In fact, marginally higher levels based on NSAF values were observed for serpin (0.24 NSAF) and stefin (0.12 NSAF) inhibitors. In situ hybridisation studies localised the KT1 gene transcript to haematin (or digestive) cells and not the digestive tract [24], which is consistent with its lower expression in the secretome.

Analysis of the most abundant proteins in the secretome of adult *E. nipponicum* revealed a predominance of proteins involved in immunomodulation, which is critical for parasite survival (Table 6). The second most abundant single protein was a peptidyl-prolyl cis-trans isomerase (1.37 NSFA), which, as previous studies have shown, is involved in the modulation of dendritic and T cell responses in other parasitic platyhelminths [56, 57]. In addition to their role in haemoglobin degradation, cathepsin L peptidases (1.11 NSFA) also play a key role in immunomodulation [21, 22, 54]. Other important abundant molecules present in the *E. niponnicum* secretome which probably play a role in the modulation/suppression of host immune response are fatty acid binding proteins (n = 3; 1.05 NSAF) and CD59-like molecules (n = 4; 1.08 NSAF) (Table 6).

**Table 6:** The most abundant individual proteins in *E. nipponicum* secretome quantified by NSAF

Protein	NSAF (%)	Function
Neuroglobin-like isoform X3	1.41	Well-characterised in vertebrates, probably alternative function to oxygen transport [55]
Peptidyl-prolyl cis-trans isomerase (cyclophilin)	1.37	Binding of immunosuppressive drugs, host immunomodulation, modulation of dendritic and T cell response [56, 57]
Cathepsin L1	1.11	Macromolecules digestion, immunomodulation [21, 22, 58]
Actin gamma	1.05	Intracellular multi-functional protein, in insect described as extracellular immune factor which interacts with bacteria [59]
Neuroglobin-like isoform X2	0.94	Described above
Peptidyl-prolyl cis-trans isomerase (cyclophilin)	0.93	Described above
SmKK7	0.74	*Schistosoma mansoni* antigen [60, 61]
Actin	0.73	Described above
Fatty acid-binding protein	0.63	Anti-inflammatory protein, inhibitor of Toll-like receptor 4 (TLR4) [62, 63]
Superoxide dismutase	0.59	Antioxidant enzyme which accelerates the dismutation of superoxide to hydrogen peroxide [64]
Annexin	0.57	Fundamental biological activities (metabolism, cell adhesion, growth, subcellular transport, membrane repair), modulation of vertebrate host immune response [65, 66]
Annexin	0.53	Described above
Actin	0.50	Described above
Glyceraldehyde-3-phosphate dehydrogenase	0.50	Inhibits complement cascade by binding to complement C3 in *Haemonchus contortus* [67, 68]
CD59-like protein	0.50	Inhibition of the complement cascade [69]

As shown above, adaptation to a blood feeding strategy leads among other things to increased levels of oxidative stress resulting from the release of iron from haemoglobin. To neutralise or reduce the levels of free radicals, the parasite produces a range of antioxidants, including superoxide dismutase (SOD; 0.70 NSAF), thioredoxin (TRX; 0.30 NSAF), and peroxiredoxin (PRX; 0.26 NSAF), which are then found in the secretome.

## Discussion

Monogeneans are the most species-rich group of fish-infecting parasites within the phylum Platyhelminthes. They evolved unique morphological adaptations associated with parasitism, including varying shape and size of their attachment organs and hooks, which are widely used for species identification. These traditional morphological methods are nowadays often combined with molecular sequencing technologies to provide more robust determination approaches [70]. On the other hand, despite the advanced methods for classifying these parasites, the amount of molecular and biochemical data pertaining to them is meagre. In this study, we conducted in-depth transcriptome and secretome analyses to further our understanding of adult *E. nipponicum* parasites, which helped us provide novel insights into this blood-feeding parasite’s feeding strategy.

*E. nipponicum* is an obligate blood-feeding ectoparasite. As a consequence, fish infected with it suffer from decreased levels of haemoglobin [71] and hypochromic microcytic anaemia, characterised by increased ratio of immature red blood cells [2]. The process of blood feeding is initiated following attachment to the fish host, where the gill tissue is mechanically damaged by pressure created by suckers located in parasite’s oral cavity, which leads to bleeding from host’s superficial capillaries. There is currently no evidence that this process is supported by peptidases secreted by the parasite that would digest the host tissue, as has been reported for mucus-feeding monogeneans which most employ use elastase-like serine peptidases to that purpose [21]. In the case of *E. nipponicum*, some proteins it secretes are involved in preventing blood coagulation and digestion in its gut lumen and specialised digestive (haematin) cells [45].

In this respect, the *E. nipponicum* resembles other blood-feeding platyhelminths which initiate haemoglobin processing extracellularly. For instance, the schistosomes digest the bloodmeal extracellularly in the gut lumen using a range of cathepsin peptidases secreted from their gastrodermis [72], while the liver fluke *Fasciola hepatica* combines extracellular digestion in the gut facilitated by cathepsin L peptidases with intracellular digestion by cathepsin C and aminopeptidases following absorption of haemoglobin peptides in its gastrodermal epithelial cells [44]. Intriguingly, the process of fully intracellular blood digestion resembles most closely the strategy of blood-feeding tick *I. ricinus* [73], which presents a clear contrast to other blood-feeding arthropods, mainly insects, that rely solely on extracellular digestion [74].

Despite the different location and use of various digestive enzymes that play a role in haemoglobin digestion, the biochemistry *E. nipponicum* digestion resembles that of other blood-feeding platyhelminths which use a range of cathepsin peptidases and peptidase inhibitors. Our analysis of adult parasite transcriptome and secretome revealed that the parasite abundantly expresses several key peptidases, including cathepsins B, D, L1, and L3, which play a critical role in haemoglobin processing [22]. We have previously shown that serine peptidase inhibitors, Kunitz-type inhibitor EnKT1, and serpin EnSerp1 target host peptidases belonging to the coagulation cascade, such as factors IIa (thrombin) and Xa [23, 24]. Our current analysis of the adult transcriptome and secretome had moreover revealed that the genes which encode these peptidase inhibitors are highly transcribed and that the abovementioned inhibitors are indeed secreted by adult parasites, although they are not present in the ESP in large quantities. This secretome analysis is consistent with our previous characterisation of the EnSerp1 [23]. Similarly, in situ hybridisation studies localised the EnKT1 gene transcript to the haematin (or digestive) cells and not the digestive tract [24], which is consistent with its low expression in the secretome.

Our analysis of the transcriptome and secretome had also revealed that ferritin proteins and GSTs play an important role in the life of adult *E. nipponicum*, in particular its iron and haem processing. Ferritins are globular proteins which store and transport iron ions in a soluble and non-toxic form. Although ferritins are essential for all blood-feeding parasites [75–79], their abundance in the *E. nipponicum* transcriptome and secretome seems more akin to the blood-feeding strategy of ticks [48]. Also abundantly expressed in the secretome, at comparable levels, are GST molecules which are part of the phase II detoxification system. Studies of blood-feeding ticks and nematodes had shown that in addition to being involved in drug metabolism, these molecules also play an essential role in haem detoxification. In ticks, gene transcription of detoxification enzymes such as the GSTs is upregulated following blood feeding [38, 49]. Similarly, it has been shown that nematodes *Haemonchus contortus* and *Caenorhabditis elegans* express Nu-class GSTs which can bind the haem [50]. Further investigation is required to determine the class and function of the *E. nipponicum* GSTs to determine what role they might play in haem detoxification.

Both free-living and parasitic nematodes have lost the ability to synthesise haem de novo, a fact reflected in the absence of haem biosynthesis pathway [38]. Platyhelminthes, on the other hand, retained the genes associated with haem biosynthesis pathway, although several parasitic platyhelminths adopted a blood-feeding strategy, which may indicate that the haem biosynthesis pathway may be of importance during nonblood-feeding stages as well [38]. There is currently little information on the potential for de novo haem biosynthesis in monogenean parasites. For instance, the *G. salaris* genome encodes all enzymes involved in this pathway [38], which is consistent with this parasite’s feeding strategy that relies on mucus and skin rather than blood. Our analysis of the *E. nipponicum* transcriptome shows that the first enzyme in the haem biosynthesis pathway, 5-aminolevulinic acid synthase (ALAS), is absent in its transcriptome. Moreover, all other enzymes belonging to this pathway are transcribed at low levels, which implies that haem biosynthesis is not active in this parasite, at least not during the adult stage. Further analysis is required to determine whether all components of the pathway are present in the *E. nipponicum* genome, whether they are differently regulated during its distinct lifecycle stages, and to test whether these enzymes are functional. Similarly, further investigation of monogeneans is required to determine whether they all use a conservatived strategy (and keep on synthetising the haem themselves) or whether some of those rely on absorption of host haem.

As an ectoparasite, *E. nipponicum* is not subjected to the same host immune response that endoparasitic platyhelminths which migrate throughout the host must deal with. Still, *E. nipponicum* must employ evasion strategies to target molecules within the host blood. Key molecules identified in the *E. nipponicum* secretome that likely play a role in the modulation/suppression of host immune response include several fatty acid-binding proteins (FABPs) and CD59-like molecules. The function of FABPs in monogeneans is currently unknown, but in *F. hepatica* these molecules play a role in fatty acid uptake from host blood and in immunomodulation, where they suppress Toll-like receptor (TLR) stimulation and signalling [62, 63, 80, 81]. CD59-like molecules are abundantly expressed in *E. nipponicum* secretome and they may play a role in modulating/inhibiting the host complement system by molecular mimicry in a fashion similar to that described in other platyhelminths [82]. It has been shown that infections with ectoparasites, such as *Ichthyophthirius multifiliis*, stimulate the expression of the carp complement system [83] and in salmonid fish, the host complement has a lethal effect on monogeneans *Gyrodactylus derjavini* and *G. salaris* [84, 85].

## Conclusions

In the present study, we explored the transcriptome and secretome of adult *E. nipponicum* worms using bioinformatic analyses of RNA-seq and LC-MS/MS data. The datasets and results we obtained are unique for parasitic monogeneans because a similarly comprehensive transcriptomic/secretomic study has not been undertaken before. The reported primary dataset can be used for further monogenean research as well as for identification of protein molecules involved in host–parasite interactions. Our insight into transcribed molecules of *E. nipponicum* revealed a machinery of highly expressed proteins critical for (a) the suppression of anticoagulation processes of the fish host by deployment of protease inhibitors (Kunitz-type inhibitors and serpins, CD59-like proteins); (b) digestion of blood proteins (cathepsins) and iron processing (ferritin), and (c) modulation of immune reaction (peptidyl-prolyl cis-trans isomerase, fatty acid binding proteins, and tetraspanin).

## Methods

### Parasite material

*E. nipponicum* adults were collected during the summer periods from the gills of naturally infected and freshly sacrificed carp (*C. carpio*) bred in the ponds of a local commercial fishery in southwestern Czech Republic (Rybářství Třeboň, Plc., Třeboň basin, South Bohemia, Czech Republic).

### Collection of excretory–secretory proteins (ESP)

ESP were collected from 100 adult worms. Worms were gently washed in sterile tap water and incubated in 10 mM PBS, pH 7.2, for three hours at room temperature in Eppendorf tubes. ESP were purified and concentrated 20 times by centrifugation using an Amicon Ultra 3 kDa column (Merck Millipore) to a final volume of 5 ml. Protein concentration (0.01 μg · μl^− 1^) was determined using Quaint-iT Protein Assay Kit (Life Technologies) and SpectraMax i3 fluorometer (Molecular Devices). The ESP sample was stored at − 80 °C until used.

### RNA extraction, library preparation, and sequencing

Total RNA was isolated from ten *E. nipponicum* adults (two independent replicates of five worms) using TriPure Isolation Reagent (Roche) according to manufacturer’s instructions. This was followed by DNase I treatment as previously described [86]. RNA concentrations were quantified spectrophotometrically (NanoDrop 8000, Thermo Fisher Scientific) and fluorometrically (Qubit 2.0, Life Technologies), and integrity was verified by gel electrophoresis using 2100 BioAnalyser (Agilent). GS FLX Titanium Rapid library was prepared from one replicate of five worms using 1.2 μg of total RNA according to manufacturer’s instructions (GS FLX Titanium Rapid library preparation kit v. 3.0, 454 Life Sciences). Illumina TruSeq RNA library (non-stranded TruSeq RNA Library Prep Kit v. 2, Illumina) was prepared from 1 μg of total RNA extracted from the second replicate of five worms as previously described [87]. The libraries were sequenced using appropriate sequencing platforms, namely GS FLX Titanium Roche 454 (single-end sequencing) and MiSeq Illumina (short-insert paired-end sequencing, 2 × 100 bp long reads). Sequencing was carried out by BGI Group, Hong Kong (Illumina sequencing) and by the Faculty of Medicine in Hradec Králové, Charles University, Czech Republic (Roche 454 sequencing).

### Processing of raw reads, de novo assembly, and annotation

The quality of raw sequencing paired-end Illumina reads, exported in FASTQ format, was evaluated using FastQC v. 0.11.3 [88]. Sequencing adaptors and nucleotides with Phred quality score below 28 were trimmed using Trimmomatic v. 0.33 [89] and sequencing errors and mismatches corrected using SPAdes v. 3.6.0 [90] (BayesHammer tool; *−-only-error-correction* and *--careful* modes). Contaminating reads from the fish host were removed using TopHat v. 2.0.14 [91] by aligning RNA-seq reads to the carp genome (NCBI Genome ID 10839). Processed reads were finally assembled by Oases v. 0.2.08 [92] with coverage cut-off ranging from 2 to 26 (increasing by one) and k-mers values ranging from 19 to 67 (increasing by two). All assembled transcriptomic datasets were statistically evaluated in the following steps: (a) Basic transcriptome assembly quality analysis was carried out by Transrate v. 1.0.3 [93]; (b) Highly conserved eukaryotic core genes were classified using CEGMA v. 2.5 [94] and BUSCO v. 3.0.1. (Metazoa dataset) [95]; (c) The raw sequencing reads were mapped to the assembled contigs by Burrows-Wheeler Aligner (BWA-backtrack algorithm) v. 0.7.13 [96], and (d) Transdecoder v. 3.0.1 [97] was used to calculate the representation of nucleotide sequences encoding a protein (from each nucleotide contig only the longest protein-coding part was selected, with minimal protein length 30 amino acids).

Read information from the SFF file generated by Roche 454 sequencer was extracted and converted into a FASTQ format using tool sff2fastq v. 0.9.2 [98]. The quality of raw reads was evaluated using FastQC v. 0.11.5 [88]. Adaptors and nucleotides with Phred quality score below 18 were discarded by Trimmomatic v. 0.36 [89] and sequencing errors corrected by Pollux v. 1.0.2 [99]. Contaminating reads from the fish host were identified by Burrows-Wheeler Aligner (BWA-SW algorithm) v. 0.7.13 [100] by mapping to the carp genome. Final assembly was performed by SPAdes v. 3.9.0 [90] (rnaSPAdes tool) based on three datasets generated from the Illumina data (k-mer value 53 and coverage cut-off value 8; k-mer value 57 and coverage cut-off value 9; k-mer value 55 and coverage cut-off value 10) and four datasets generated from the Roche 454 reads (k-mer values 99, 95, 87, and 83). Statistical evaluation of assemblies was performed as above. Duplicate sequences were removed after clustering using CD-HIT-EST (nucleotide identity threshold 95%) [101]. The final transcript dataset used for further analysis was based on sequences representing the longest open reading frames encoding at least 30 amino acids, which were selected by Transdecoder.

Annotation of the *E. nipponicum* transcripts was carried out by BLAST analysis with a cut-off of 1e^− 5^ (v. 2.7.1 [102];) using the following databases: (a) NCBI non-redundant protein database [103]; (b) MEROPS database of peptidases and their inhibitors [104]; (c) UniProtKB/UniRef100 database; (d) UniProtKB/TrEMBL database, which only includes sequences related to the Platyhelminthes (Taxon 6157) [105]; (e) UniProtKB/Swiss-Prot [106]; (f) RCSB PDB database of proteins with known structure [107]; (g) nucleotide DDBJ database [108].

All transcripts with high sequence similarity to potential contaminating sequences (virus, bacteria, cyanobacteria, yeast, fungi, algae, green plants, or carp) were removed from the dataset prior to further analysis. Additionally, we also excluded any sequences with a potential open reading frame of less than 50 amino acids with no putative annotation. Further in silico analysis was conducted using the following tools: (a) *KAAS (KEGG Automatic Annotation Server* [34, 109]*); (b)* Gene Ontology (GO) prediction was performed using InterProScan v. 5.30–69.0 [110] with default search parameters; (c) transcript abundance was quantified using RSEM v. 1.3.1 [111] by mapping trimmed and corrected Roche 454 and Illumina reads onto the final transcriptome. The resulting TPM values were averaged, which was necessitated by the varied character of sequential data (single-end and paired-end), while Roche 454 and Illumina reads have to be mapped separately. Transcription abundance was measured by the sum of the TPM values of all the participating transcripts in a given set. Additionally, we calculated a ratio between the TPM value and the number of participating transcripts was calculated to provide information regarding how many transcripts were responsible for transcription abundance. GO terms and KEGG pathways with less than 10 transcripts were excluded from this analysis.

### Identification of the excretory–secretory proteins by mass spectrometry

ESP sample was digested using filter-aided sample preparation with 1 μg of trypsin (sequencing grade, Promega). For peptide separation for MS/MS analysis, we used UltiMat 3000 RSLCnano liquid chromatography (LC) system (Thermo Fisher Scientific). Separation was achieved using a capillary column filled with the nonpolar stationary phase (500 mm × 75 μm, C18 anchors, 3 μm particles, Acclaim PepMap, Thermo Fisher Scientific) during a 135 min gradient elution (0.5 μl · min^− 1^). Mobile phase consisted of a polar (0.1% formic acid (FA)) and nonpolar phase (80% acetonitrile (ACN), 0.1% FA). Eluted peptides (2 μg) were ionised by a nanospray (PicoView 550 nano source) and analysed in a mass spectrometer (Orbitrap Elite, Thermo Fisher Scientific). MS data were acquired in a data-dependent mode, selecting up to top ten precursors based on precursor abundance in the survey scan (resolution 60,000 in the range 350–2000 m/z). Maximum accumulation time for MS/MS spectra acquisition was 500 ms (resolution 15,000 at 400 m/z) and isolation window for fragmentation was set to 2 m/z. The resulting MS data were recalibrated using 445.120028 signal from the first 10 min and used for identification of proteins. Mass spectrometric raw data files were analysed using Proteome Discoverer software (Thermo Fisher Scientific; v. 1.4) with in-house Mascot search engine (Matrixscience; v. 2.5.1.3) set up to search an in-house protein database containing 37,062 protein sequences derived from the *E. nipponicum* transcriptome sequencing, carp-specific proteins derived from *C. carpio* genome (63,928 sequences, NCBI Genome 10839), and cRAP contaminants (110 sequences). Modifications for all database searches were set as follows: oxidation (M), deamidation (N, Q), and acetylation (Protein N-term) as variable modifications, with carbamidomethylation (C) as a fixed modification. Enzyme specificity was semitryptic with one allowed miscleavage. Percolator was used for postprocessing of search results. Only peptides with q-value < 0.05, rank 1, and with at least six amino acids were considered. LC-MS/MS analysis was conducted at Proteomics Core Facility, CEITEC, Masaryk University, Czech Republic.

## Supplementary Information

**Additional file 1: Table S1.** Spreadsheet with a list of *E. nipponicum* transcripts with nucleotide and amino acid sequences and their lengths, sequence completeness, and information about the presence of signal peptide.

**Additional file 2: Table S2.** Spreadsheet representing and summarising all results from the annotation of the *E. nipponicum* transcriptome (outputs of BLAST analyses), including outputs of KASS and InterPro functional analyses, GO terms, and TPM values.

**Additional file 3: Table S3.** Spreadsheet presenting the most abundant *E. nipponicum* transcripts quantified by TPM value.

**Additional file 4: Table S4.** Spreadsheet with a list of observed *E. nipponicum* GO terms. Each GO term is described (annotated), sorted into one of three main categories, and quantified by the sum of TPM values and the number of included transcripts and the ratio between the TPM value and the number of transcripts.

**Additional file 5: Table S5.** Spreadsheet with observed *E. nipponicum* KEGG Modules (by KAAS analysis) with information about the total number of transcripts in each module and the total of TPM and the ratio between the TPM value and the number of transcripts.

**Additional file 6: Table S6.** Spreadsheet presenting *E. nipponicum* KEGG Orthologues and pathways. Pathways are linked with K number and quantified by the sum of TPM values of all included transcripts (also presented) and the ratio between the TPM value and the number of transcripts.

**Additional file 7: Table S7.** Spreadsheet with a list of *E. nipponicum* secreted proteins (ESP) identified by LC-MS/MS analysis. Additional information for each protein is molecular weight, pI value, area, coverage, number of detected peptides (total, unique), quantification (SAF and NSAF), and annotation.

## Data Availability

This Transcriptome Shotgun Assembly project was deposited at DDBJ/ENA/GenBank under the accession GFYM00000000. The version described in this paper is the first version, GFYM01000000. Raw sequence data were deposited in the NCBI SRA database under accession numbers SRX2995121 (Roche 454) and SRX2995122 (Illumina). Mass spectrometry proteomics data were deposited to the ProteomeXchange Consortium via the PRIDE partner repository under dataset identifier PXD017293. Transcriptomic sequences and annotation are available on GitHub repository Eudiplozoon-nipponicum-transcriptome-secretome (https://github.com/jirivorel/Eudiplozoon-nipponicum-transcriptome-secretome).

## Abbreviations

BLAST: Basic Local Alignment Search Tool
bp: base pair
DDBJ: DNA Data Bank of Japan
ESP: excretory–secretory proteins
EST: expressed sequence tag
EVs: extracellular vesicles
FABPs: fatty acid binding proteins
GO: Gene Ontology
GSTs: glutathione S-transferases
KAAS: KEGG Automatic Annotation Server
KEGG: Kyoto Encyclopedia of Genes and Genomes
KO: KEGG orthology
LC-MS/MS: liquid chromatography – tandem mass spectrometry
NCBI: National Centre for Biotechnology Information
NSFA: normalised spectral abundance factor
SRA: Sequence Read Archive
TLR: toll-like receptor
TPM: transcripts per million

## References

[1] Ogawa K (2014). Diseases of cultured marine fishes caused by Platyhelminthes (Monogenea, Digenea, Cestoda). Parasitology..

[2] Kawatsu H (1978). Studies on the anemia of fish-IX. Nippon Suisan Gakkaishi.

[3] Bakke TA, Harris PD, Hansen H, Cable J, Hansen LP (2004). Susceptibility of Baltic and East Atlantic salmon *Salmo salar* stocks to *Gyrodactylus salaris* (Monogenea). Dis Aquat Org.

[4] Huyse T, Plaisance L, Webster BL, Mo TA, Bakke TA, Bachmann L (2006). The mitochondrial genome of *Gyrodactylus salaris* (Platyhelminthes: Monogenea), a pathogen of Atlantic salmon (*Salmo salar)*. Parasitology..

[5] Plaisance L, Huyse T, Littlewood DTJ, Bakke TA, Bachmann L (2007). The complete mitochondrial DNA sequence of the monogenean *Gyrodactylus thymalli* (Platyhelminthes: Monogenea), a parasite of grayling (*Thymallus thymallus*). Mol Biochem Parasitol.

[6] Zhang J, Wu X, Li Y, Zhao M, Xie M, Li A (2014). The complete mitochondrial genome of *Neobenedenia melleni* (Platyhelminthes: Monogenea): mitochondrial gene content, arrangement and composition compared with two *Benedenia* species. Mol Biol Rep.

[7] Kang S, Kim J, Lee J, Kim S, Min G-S, Park J-K (2012). The complete mitochondrial genome of an ectoparasitic monopisthocotylean fluke *Benedenia hoshinai* (Monogenea: Platyhelminthes). Mitochondrial DNA.

[8] Zhang J, Wu X, Xie M, Li A (2012). The complete mitochondrial genome of *Pseudochauhanea macrorchis* (Monogenea: Chauhaneidae) revealed a highly repetitive region and a gene rearrangement hot spot in Polyopisthocotylea. Mol Biol Rep.

[9] Hahn C, Fromm B, Bachmann L (2014). Comparative genomics of flatworms (Platyhelminthes) reveals shared genomic features of ecto- and endoparastic neodermata. Genome Biol Evol..

[10] Konczal M, Przesmycka KJ, Mohammed RS, Phillips KP, Camara F, Chmielewski S, Hahn C, Guigo R, Cable J, Radwan J (2020). Gene duplications, divergence and recombination shape adaptive evolution of the fish ectoparasite *Gyrodactylus bullatarudis*. Mol Ecol.

[11] Coghlan A, Tyagi R, Cotton JA, Holroyd N, Rosa BA, Tsai I (2019). Comparative genomics of the major parasitic worms. Nat Genet.

[12] Roudnický P, Potěšil D, Zdráhal Z, Gelnar M, Kašný M (2020). Laser capture microdissection in combination with mass spectrometry: approach to characterisation of tissue-specific proteomes of *Eudiplozoon nipponicum* (Monogenea, Polyopisthocotylea). PLoS One.

[13] Valigurová A, Hodová I, Sonnek R, Koubková B, Gelnar M (2011). *Eudiplozoon nipponicum* in focus: monogenean exhibiting a highly specialised adaptation for ectoparasitic lifestyle. Parasitol Res.

[14] Hodová I, Matějusová I, Gelnar M (2010). The surface topography of *Eudiplozoon nipponicum* (Monogenea) developmental stages parasitising carp (*Cyprinus carpio* L.). Cent Eur J Biol.

[15] Denis A, Gabrion C, Lambert A (1983). Présence en France de deux parasites d’origine est-asiatique : *Diplozoon nipponicum* Goto, 1891 (Monogenea) et *Bothriocephalus acheilognathi* Yamaguti, 1934 (Cestoda) chez *Cyprinus carpio* (Teleostei, Cyprinidae). Bull Fr Piscic.

[16] Matějusová I, Koubková B, D’Amelio S, Cunningham CO (2001). Genetic characterisation of six species of diplozoids (Monogenea; Diplozoidae). Parasitology..

[17] The Food and Agriculture Organization of the United Nations, Fisheries Division. FishStatJ - Software for Fishery and Aquaculture Statistical Time Series. Available from http://www.fao.org/fishery/statistics/software/fishstatj/en. Accessed 10 December 2020.

[18] Matějusová I, Koubková B, Cunningham CO (2004). Identification of European diplozoids (Monogenea, Diplozoinae) by restriction digestion of the ribosomal RNA internal transcribed spacer. J Parasitol.

[19] Košková E, Matějusová I, Civáňová K, Koubková B (2010). Ethanol-fixed material used for both classical and molecular identification purposes: *Eudiplozoon nipponicum* (Monogenea: Diplozoidae) as a case parasite species. Parasitol Res.

[20] Ilgová J, Jedličková L, Dvořáková H, Benovics M, Mikeš L, Janda L, Vorel J, Roudnický P, Potěšil D, Zdráhal Z, Gelnar M, Kašný M (2017). A novel type I cystatin of parasite origin with atypical legumain-binding domain. Sci Rep.

[21] Jedličková L, Dvořáková H, Dvořák J, Kašný M, Ulrychová L, Vorel J, Žárský V, Mikeš L (2018). Cysteine peptidases of *Eudiplozoon nipponicum*: a broad repertoire of structurally assorted cathepsins L in contrast to the scarcity of cathepsins B in an invasive species of haematophagous monogenean of common carp. Parasites Vectors.

[22] Jedličková L, Dvořáková H, Kašný M, Ilgová J, Potěšil D, Zdráhal Z (2016). Major acid endopeptidases of the blood-feeding monogenean *Eudiplozoon nipponicum* (Heteronchoinea: Diplozoidae). Parasitology..

[23] Roudnický P, Vorel J, Ilgová J, Benovics M, Norek A, Jedličková L, Mikeš L, Potěšil D, Zdráhal Z, Dvořák J, Gelnar M, Kašný M (2018). Identification and partial characterisation of a novel serpin from *Eudiplozoon nipponicum* (Monogenea, Polyopisthocotylea). Parasite..

[24] Jedličková L, Dvořák J, Hrachovinová I, Ulrychová L, Kašný M, Mikeš L (2019). A novel Kunitz protein with proposed dual function from *Eudiplozoon nipponicum* (Monogenea) impairs haemostasis and action of complement in vitro. Int J Parasitol.

[25] Ilgová J, Kavanová L, Matiašková K, Salát J, Kašný M (2020). Effect of cysteine peptidase inhibitor of *Eudiplozoon nipponicum* (Monogenea) on cytokine expression of macrophages *in vitro*. Mol Biochem Parasitol.

[26] Nishihira T, Urabe M (2020). Morphological and molecular studies of *Eudiplozoon nipponicum* (Goto, 1891) and *Eudiplozoon kamegaii* sp. n. (Monogenea; Diplozoidae). Folia Parasitol.

[27] Chmúrčiaková N, Kašný M, Orosová M (2020). Cytogenetics of *Eudiplozoon nipponicum* (Monogenea, Diplozoidae): karyotype, spermatocyte division and 18S rDNA location. Parasitol Int.

[28] Konstanzová V, Koubková B, Kašný M, Ilgová J, Dzika E, Gelnar M (2016). Excretory system of representatives from family Diplozoidae (Monogenea). Parasitol Res.

[29] Schabussova I, Koubková B, Gelnar M, Schabuss M, Horák P (2004). Surface carbohydrates of *Eudiplozoon nipponicum* pre- and post-fusion. J Helminthol.

[30] Zurawski TH, Mousley A, Mair GR, Brennan GP, Maule AG, Gelnar M, Halton DW (2001). Immunomicroscopical observations on the nervous system of adult *Eudiplozoon nipponicum* (Monogenea: Diplozoidae). Int J Parasitol.

[31] Zurawski TH, Mousley A, Maule AG, Gelnar M, Halton DW (2003). Cytochemical studies of the neuromuscular systems of the diporpa and juvenile stages of *Eudiplozoon nipponicum* (Monogenea: Diplozoidae). Parasitology..

[32] Zurawski TH, Mair GR, Maule AG, Gelnar M, Halton DW (2003). Microscopical evaluation of neural connectivity between paired stages of *Eudiplozoon nipponicum* (Monogenea: Diplozoidae). J Parasitol.

[33] Coakley G, Maizels RM, Buck AH (2015). Exosomes and other extracellular vesicles: the new communicators in parasite infections. Trends Parasitol.

[34] Kanehisa M, Goto S (2000). KEGG: Kyoto encyclopedia of genes and genomes. Nucleic Acids Res.

[35] Halton DW, Stranock SD, Hardcastle A (1974). Vitelline cell development in monogenean parasites. Z Parasitenkd.

[36] Dvořák J, Horn M (2018). Serine proteases in schistosomes and other trematodes. Int J Parasitol.

[37] Chaimon S, Limpanont Y, Reamtong O, Ampawong S, Phuphisut O, Chusongsang P, Ruangsittichai J, Boonyuen U, Watthanakulpanich D, O’Donoghue AJ, Caffrey CR, Adisakwattana P (2019). Molecular characterisation and functional analysis of the *Schistosoma mekongi* Ca2+−dependent cysteine protease (calpain). Parasit Vectors.

[38] Perner J, Gasser RB, Oliveira PL, Kopáček P (2019). Haem biology in metazoan parasites – ‘The bright side of haem’. Trends Parasitol.

[39] Ponka P (1999). Cell biology of heme. Am J Med Sci.

[40] Heinemann IU, Jahn M, Jahn D (2008). The biochemistry of heme biosynthesis. Arch Biochem Biophys.

[41] Reddi AR, Hamza I (2016). Heme mobilization in animals: a metallolipid’s journey. Acc Chem Res.

[42] Sonenshine DE, Roe RM (2013). Biology of ticks, volume 1.

[43] Smyth JD, Halton DW, Smyth JD, Halton DW (1983). The Monogenean – physiology. The physiology of Trematodes.

[44] Robinson MW, Dalton JP, Donnelly S (2008). Helminth pathogen cathepsin proteases: it’s a family affair. Trends Biochem Sci.

[45] Konstanzová V, Koubková B, Kašný M, Ilgová J, Dzika E, Gelnar M (2015). Ultrastructure of the digestive tract of *Paradiplozoon homoion* (Monogenea). Parasitol Res.

[46] Toh SQ, Gobert GN, Martínez DM, Jones MK (2015). Haem uptake is essential for egg production in the haematophagous blood fluke of humans, *Schistosoma mansoni*. FEBS J.

[47] Glanfield A, McManus DP, Anderson GJ, Jones MK (2007). Pumping iron: a potential target for novel therapeutics against schistosomes. Trends Parasitol.

[48] Galay RL, Aung KM, Umemiya-Shirafuji R, Maeda H, Matsuo T, Kawaguchi H, et al. Multiple ferritins are vital to successful blood feeding and reproduction of the hard tick *Haemaphysalis longicornis*. J Exp Biol. 2013;216(10):1905–15. 10.1242/jeb.081240. 10.1242/jeb.081240 23393286

[49] Perner J, Kotál J, Hatalová T, Urbanová V, Bartošová-Sojková P, Brophy PM, et al. Inducible glutathione S-transferase (IrGST1) from the tick *Ixodes ricinus* is a haem-binding protein. Insect Biochem Mol Biol. 2018;95:44–54. 10.1016/j.ibmb.2018.02.002. 10.1016/j.ibmb.2018.02.002 29526768

[50] Perally S, Lacourse EJ, Campbell AM, Brophy PM (2008). Heme transport and detoxification in nematodes: subproteomics evidence of differential role of glutathione transferases. J Proteome Res.

[51] Wang Q, Da’dara AA, Skelly PJ (2017). The human blood parasite *Schistosoma mansoni* expresses extracellular tegumental calpains that cleave the blood clotting protein fibronectin. Sci Rep.

[52] Sojka D, Franta Z, Frantová H, Bartošová P, Horn M, Váchová J, et al. Characterization of gut-associated cathepsin D hemoglobinase from tick *Ixodes ricinus* (IrCD1). J Biol Chem. 2012;287(25):21152–63. 10.1074/jbc.M112.347922. 10.1074/jbc.M112.347922 PMC3375538 22539347

[53] Grams R, Adisakwattana P, Ritthisunthorn N, Eursitthichai V, Vichasri-Grams S, Viyanant V (2006). The saposin-like proteins 1, 2, and 3 of *Fasciola gigantica*. Mol Biochem Parasitol.

[54] McCarthy E, Stack C, Donnelly SM, Doyle S, Mann VH, Brindley PJ, et al. Leucine aminopeptidase of the human blood flukes, *Schistosoma mansoni* and *Schistosoma japonicum*. Int J Parasitol. 2004;34(6):703–14. 10.1016/j.ijpara.2004.01.008. 10.1016/j.ijpara.2004.01.008 15111092

[55] Smith HL, Pavasovic A, Surm JM, Phillips MJ, Prentis PJ (2018). Evidence for a large expansion and subfunctionalization of globin genes in sea anemones. Genome Biol Evol.

[56] Bell A, Monaghan P, Page AP (2006). Peptidyl-prolyl cis-trans isomerases (immunophilins) and their roles in parasite biochemistry, host-parasite interaction and antiparasitic drug action. Int J Parasitol.

[57] Floudas A, Cluxton CD, Fahel J, Khan AR, Saunders SP, Amu S, et al. Composition of the *Schistosoma mansoni* worm secretome: identification of immune modulatory Cyclophilin a. PLoS Negl Trop Dis. 2017;11(10):e0006012. 10.1371/journal.pntd.0006012. 10.1371/journal.pntd.0006012 PMC5681295 29073139

[58] Dalton JP, Robinson MW, Mulcahy G, O’Neill SM, Donnelly S. Immunomodulatory molecules of *Fasciola hepatica*: candidates for both vaccine and immunotherapeutic development. Vet Parasitol. 2013;195(3-4):272–85. 10.1016/j.vetpar.2013.04.008. 10.1016/j.vetpar.2013.04.008 23623183

[59] Sandiford SL, Dong Y, Pike A, Blumberg BJ, Bahia AC, Dimopoulos G (2015). Cytoplasmic actin is an extracellular insect immune factor which is secreted upon immune challenge and mediates phagocytosis and direct killing of bacteria, and is a Plasmodium antagonist. PLoS Pathog.

[60] Reimers N, Homann A, Höschler B, Langhans K, Wilson RA, Pierrot C, et al. Drug-induced exposure of *Schistosoma mansoni* antigens SmCD59a and SmKK7. PLoS Negl Trop Dis. 2015;9(3):e0003593. 10.1371/journal.pntd.0003593. 10.1371/journal.pntd.0003593 PMC4361651 25774883

[61] Egesa M, Lubyayi L, Jones FM, van Diepen A, Chalmers IW, Tukahebwa EM, et al. Antibody responses to *Schistosoma mansoni* schistosomula antigens. Parasite Immunol. 2018;40(12):e12591. 10.1111/pim.12591. 10.1111/pim.12591 PMC6492298 30239012

[62] Ramos-Benítez MJ, Ruiz-Jiménez C, Aguayo V, Espino AM (2017). Recombinant *Fasciola hepatica* fatty acid binding protein suppresses toll-like receptor stimulation in response to multiple bacterial ligands. Sci Rep.

[63] Martin I, Cabán-Hernández K, Figueroa-Santiago O, Espino AM (2015). *Fasciola hepatica* fatty acid binding protein inhibits TLR4 activation and suppresses the inflammatory cytokines induced by lipopolysaccharide *in vitro* and *in vivo*. J Immunol.

[64] Chiumiento L, Bruschi F (2009). Enzymatic antioxidant systems in helminth parasites. Parasitol Res.

[65] Tararam CA, Farias LP, Wilson RA, Leite LC de C. (2010). *Schistosoma mansoni* Annexin 2: molecular characterisation and immunolocalisation. Exp Parasitol.

[66] Yan H-L, Xue G, Mei Q, Ding F-X, Wang Y-Z, Sun S-H. Calcium-dependent proapoptotic effect of *Taenia solium* metacestodes annexin B1 on human eosinophils: a novel strategy to prevent host immune response. Int J Biochem Cell Biol. 2008;40(10):2151–63. 10.1016/j.biocel.2008.02.018. 10.1016/j.biocel.2008.02.018 18378486

[67] Sahoo S, Murugavel S, Devi IK, Vedamurthy GV, Gupta SC, Singh BP, Joshi P (2013). Glyceraldehyde-3-phosphate dehydrogenase of the parasitic nematode *Haemonchus contortus* binds to complement C3 and inhibits its activity. Parasite Immunol.

[68] Rajan P, Mishra PKK, Joshi P (2019). Defining the complement C3 binding site and the antigenic region of *Haemonchus contortus* GAPDH. Parasite Immunol.

[69] Wilson RA, Wright JM, de Castro-Borges W, Parker-Manuel SJ, Dowle AA, Ashton PD, et al. Exploring the *Fasciola hepatica* tegument proteome. Int J Parasitol. 2011;41(13-14):1347–59. 10.1016/j.ijpara.2011.08.003. 10.1016/j.ijpara.2011.08.003 22019596

[70] Benovics M, Desdevises Y, Vukić J, Šanda R, Šimková A (2018). The phylogenetic relationships and species richness of host-specific *Dactylogyrus* parasites shaped by the biogeography of Balkan cyprinids. Sci Rep.

[71] Rohlenová K, Morand S, Hyršl P, Tolarová S, Flajšhans M, Šimková A (2011). Are fish immune systems really affected by parasites? An immunoecological study of common carp (*Cyprinus carpio*). Parasit Vectors.

[72] Caffrey CR, McKerrow JH, Salter JP, Sajid M (2004). Blood “n” guts: an update on schistosome digestive peptidases. Trends Parasitol.

[73] Horn M, Nussbaumerová M, Šanda M, Kovářová Z, Srba J, Franta Z, Sojka D, Bogyo M, Caffrey CR, Kopáček P, Mareš M (2009). Hemoglobin digestion in blood-feeding ticks: mapping a multipeptidase pathway by functional proteomics. Chem Biol.

[74] Holtof M, Lenaerts C, Cullen D, Vanden BJ (2019). Extracellular nutrient digestion and absorption in the insect gut. Cell Tissue Res.

[75] Jones MK, McManus DP, Sivadorai P, Glanfield A, Moertel L, Belli SI (2007). Tracking the fate of iron in early development of human blood flukes. Int J Biochem Cell Biol.

[76] Dietzel J, Hirzmann J, Preis D, Symmons P, Kunz W (1992). Ferritins of *Schistosoma mansoni*: sequence comparison and expression in female and male worms. Mol Biochem Parasitol.

[77] Glanfield A, Mcmanus DP, Smyth DJ, Lovas EM, Loukas A, Gobert GN (2010). A cytochrome b561 with ferric reductase activity from the parasitic blood fluke, *Schistosoma japonicum*. PLoS Negl Trop Dis.

[78] Cabán-Hernández K, Gaudier JF, Espino AM (2012). Characterization and differential expression of a ferritin protein from *Fasciola hepatica*. Mol Biochem Parasitol.

[79] Tang Y, Cho PY, Kim TI, Hong S-J (2006). *Clonorchis sinensis*: molecular cloning, enzymatic activity, and localization of yolk ferritin. J Parasitol.

[80] Figueroa-Santiago O, Espino AM (2014). *Fasciola hepatica* fatty acid binding protein induces the alternative activation of human macrophages. Infect Immun.

[81] Morphew RM, Wilkinson TJ, MacKintosh N, Jahndel V, Paterson S, McVeigh P (2016). Exploring and expanding the fatty-acid-binding protein superfamily in *Fasciola* species. J Proteome Res.

[82] Shao S, Sun X, Chen Y, Zhan B, Zhu X (2019). Complement evasion: an effective strategy that parasites utilise to survive in the host. Front Microbiol.

[83] Gonzalez SF, Buchmann K, Nielsen ME (2007). Complement expression in common carp (*Cyprinus carpio* L.) during infection with *Ichthyophthirius multifiliis*. Dev Comp Immunol.

[84] Buchmann K (1998). Binding and lethal effect of complement from *Oncorhynchus mykiss* on Gyrodactylus derjavini (Platyhelminthes: Monogenea). Dis Aquat Org.

[85] Harris PD, Soleng A, Bakke TA (1998). Killing of *Gyrodactylus salaris* (Platyhelminthes, Monogenea) mediated by host complement. Parasitology..

[86] Young ND, Jex AR, Cantacessi C, Hall RS, Campbell BE, Spithill TW, Tangkawattana S, Tangkawattana P, Laha T, Gasser RB (2011). A portrait of the transcriptome of the neglected trematode, *Fasciola gigantica* - biological and biotechnological implications. PLoS Negl Trop Dis.

[87] Cantacessi C, Mulvenna J, Young ND, Kašný M, Horaká P, Aziz A (2012). A deep exploration of the transcriptome and “excretory/secretory” proteome of adult *Fascioloides magna*. Mol Cell Proteomics.

[88] FastQC. A quality control tool for high throughput sequence data. Available from https://www.bioinformatics.babraham.ac.uk/projects/fastqc/. Accessed 10 December 2020.

[89] Bolger AM, Lohse M, Usadel B (2014). Trimmomatic: a flexible trimmer for Illumina sequence data. Bioinformatics..

[90] Bankevich A, Nurk S, Antipov D, Gurevich AA, Dvorkin M, Kulikov AS, Lesin VM, Nikolenko SI, Pham S, Prjibelski AD, Pyshkin AV, Sirotkin AV, Vyahhi N, Tesler G, Alekseyev MA, Pevzner PA (2012). SPAdes: a new genome assembly algorithm and its applications to single-cell sequencing. J Comput Biol.

[91] Trapnell C, Pachter L, Salzberg SL (2009). TopHat: discovering splice junctions with RNA-Seq. Bioinformatics..

[92] Schulz MH, Zerbino DR, Vingron M, Birney E (2012). Oases: robust de novo RNA-seq assembly across the dynamic range of expression levels. Bioinformatics..

[93] Smith-Unna R, Boursnell C, Patro R, Hibberd JM, Kelly S (2016). TransRate: reference-free quality assessment of de novo transcriptome assemblies. Genome Res.

[94] Parra G, Bradnam K, Korf I (2007). CEGMA: a pipeline to accurately annotate core genes in eukaryotic genomes. Bioinformatics..

[95] Waterhouse RM, Seppey M, Simão FA, Manni M, Ioannidis P, Klioutchnikov G, Kriventseva EV, Zdobnov EM (2018). BUSCO applications from quality assessments to gene prediction and phylogenomics. Mol Biol Evol.

[96] Li H, Durbin R (2009). Fast and accurate short read alignment with burrows-wheeler transform. Bioinformatics..

[97] Haas BJ, Papanicolaou A, Yassour M, Grabherr M, Blood PD, Bowden J, Couger MB, Eccles D, Li B, Lieber M, MacManes MD, Ott M, Orvis J, Pochet N, Strozzi F, Weeks N, Westerman R, William T, Dewey CN, Henschel R, LeDuc RD, Friedman N, Regev A (2013). De novo transcript sequence reconstruction from RNA-seq using the trinity platform for reference generation and analysis. Nat Protoc.

[98] sff2fastq. Available from https://github.com/indraniel/sff2fastq. Accessed 10 December 2020.

[99] Marinier E, Brown DG, McConkey BJ (2015). Pollux: platform independent error correction of single and mixed genomes. BMC Bioinformatics..

[100] Li H, Durbin R (2010). Fast and accurate long-read alignment with burrows-wheeler transform. Bioinformatics..

[101] Li W, Godzik A (2006). Cd-hit: a fast program for clustering and comparing large sets of protein or nucleotide sequences. Bioinformatics..

[102] Altschul SF, Gish W, Miller W, Myers EW, Lipman DJ (1990). Basic local alignment search tool. J Mol Biol.

[103] Agarwala R, Barrett T, Beck J, Benson DA, Bollin C, Bolton E (2018). Database resources of the National Center for biotechnology information. Nucleic Acids Res.

[104] Rawlings ND, Barrett AJ, Finn R (2016). Twenty years of the MEROPS database of proteolytic enzymes, their substrates and inhibitors. Nucleic Acids Res.

[105] Bateman A, Martin M-J, Orchard S, Magrane M, Agivetova R, Ahmad S, et al. UniProt: the universal protein knowledgebase. Nucleic Acids Res. 2020:gkaa1100. 10.1093/nar/gkaa1100. 10.1093/nar/gkaa1100 PMC7778908 33237286

[106] Boutet E, Lieberherr D, Tognolli M, Schneider M, Bansal P, Bridge AJ (2016). UniProtKB/Swiss-Prot, the manually annotated section of the UniProt KnowledgeBase: how to use the entry view. Methods Mol Biol.

[107] Berman HM, Westbrook J, Feng Z, Gilliland G, Bhat TN, Weissig H, et al. The protein data bank. Nucleic Acids Res. 2000;28(1):235–42. 10.1093/nar/28.1.235. 10.1093/nar/28.1.235 PMC102472 10592235

[108] Mashima J, Kodama Y, Fujisawa T, Katayama T, Okuda Y, Kaminuma E, et al. DNA data bank of Japan. Nucleic Acids Res. 2017;45(D1):25–31. 10.1093/nar/gkw1001. 10.1093/nar/gkw1001 PMC5210514 27924010

[109] Moriya Y, Itoh M, Okuda S, Yoshizawa AC, Kanehisa M (2007). KAAS: an automatic genome annotation and pathway reconstruction server. Nucleic Acids Res.

[110] Jones P, Binns D, Chang H-Y, Fraser M, Li W, McAnulla C, et al. InterProScan 5: genome-scale protein function classification. Bioinformatics. 2014;30(9):1236–40. 10.1093/bioinformatics/btu031. 10.1093/bioinformatics/btu031 PMC3998142 24451626

[111] Li B, Dewey CN (2011). RSEM: accurate transcript quantification from RNA-Seq data with or without a reference genome. BMC Bioinformatics.

